# Supplementary material for: Troll story: The dark tetrad and online trolling revisited with a glance at humor
Source: PLoS One. 2023 Mar 10;18(3):e0280271. doi: 10.1371/journal.pone.0280271 (PMC10004561; doi:10.1371/journal.pone.0280271)
Supplement: S1 Appendix — (DOCX) [file pone.0280271.s001.docx]

Troll Story: Appendix

Confirmatory Factor Analysis

We conducted confirmatory factor analyses (CFAs) for all measures using the R-package “lavaan”. We primarily did so, because we adapted several measures and because our measure of immediate trolling motivation (IAIT) showed a relatively low internal consistency. CFA provides more information about how the items explain the latent factor’s variance. We used the MLR estimator for all CFAs.

| **Table 1.**  *Confirmatory Factor Analyses for our Measures.* | | | | | | |
| --- | --- | --- | --- | --- | --- | --- |
| Measure | Items | Standardized loading | *p* | CFI | RMSEA | SRMR |
| GAIT | GAIT1 | .60 |  | .89 | .08 | .05 |
|  | GAIT2 | .53 | < .001 |  |  |  |
|  | GAIT3 | .63 | < .001 |  |  |  |
|  | GAIT4 | .72 | < .001 |  |  |  |
|  | GAIT5 | .40 | < .001 |  |  |  |
|  | GAIT6 (R) | .11 | < .001 |  |  |  |
|  | GAIT7 | .37 | < .001 |  |  |  |
|  | GAIT8 | .34 | < .001 |  |  |  |
|  |  |  |  |  |  |  |
| IAIT | IAIT1 | .79 |  | .96 | .08 | .04 |
|  | IAIT2 | .84 | < .001 |  |  |  |
|  | IAIT3 | .84 | < .001 |  |  |  |
|  | IAIT4 | .63 | < .001 |  |  |  |
|  | IAIT5 | .37 | < .001 |  |  |  |
|  | IAIT7 | .27 | < .001 |  |  |  |
|  | IAIT8 | .54 | < .001 |  |  |  |
|  |  |  |  |  |  |  |
| Machiavellianism | MACH1 | .25 |  | .86 | .09 | .06 |
|  | MACH2 | .65 | < .001 |  |  |  |
|  | MACH3 | .58 | < .001 |  |  |  |
|  | MACH4 | .36 | < .001 |  |  |  |
|  | MACH5 | .73 | < .001 |  |  |  |
|  | MACH6 | .65 | < .001 |  |  |  |
|  | MACH7 | .52 | < .001 |  |  |  |
|  | MACH8 | .42 | < .001 |  |  |  |
|  | MACH9 | .39 | < .001 |  |  |  |
|  |  |  |  |  |  |  |
| Narcissism | NARC1 | .65 |  | .85 | .09 | .06 |
|  | NARC2 (R) | .54 | < .001 |  |  |  |
|  | NARC3 | .55 | < .001 |  |  |  |
|  | NARC4 | .60 | < .001 |  |  |  |
|  | NARC5 | .50 | < .001 |  |  |  |
|  | NARC6 (R) | .32 | < .001 |  |  |  |
|  | NARC7 | .38 | < .001 |  |  |  |
|  | NARC8 (R) | .52 | < .001 |  |  |  |
|  | NARC9 | .30 | < .001 |  |  |  |
| Psychopathy | PSYCH1 | .54 |  | .91 | .07 | .05 |
|  | PSYCH2 (R) | .24 | < .001 |  |  |  |
|  | PSYCH3 | .58 | < .001 |  |  |  |
|  | PSYCH4 | .52 | < .001 |  |  |  |
|  | PSYCH5 | .63 | < .001 |  |  |  |
|  | PSYCH6 | .64 | < .001 |  |  |  |
|  | PSYCH7 (R) | .26 | < .001 |  |  |  |
|  | PSYCH8 | .40 | < .001 |  |  |  |
|  | PSYCH9 | .53 | < .001 |  |  |  |
| Sadism | CAST1 | .54 |  | .68 | .08 | .09 |
|  | CAST2 | .54 | < .001 |  |  |  |
|  | CAST3 | .63 | < .001 |  |  |  |
|  | CAST4 | .55 | < .001 |  |  |  |
|  | CAST5 | .54 | < .001 |  |  |  |
|  | CAST6 (R) | .31 | < .001 |  |  |  |
|  | CAST7 | .46 | < .001 |  |  |  |
|  | CAST8 | .42 | < .001 |  |  |  |
|  | CAST9 | .40 | < .001 |  |  |  |
|  | CAST10 | .53 | < .001 |  |  |  |
|  | CAST11 | .43 | < .001 |  |  |  |
|  | CAST12 | .61 | < .001 |  |  |  |
|  | CAST13 | .66 | < .001 |  |  |  |
|  | CAST14 | .60 | < .001 |  |  |  |
|  | CAST15 | .41 | < .001 |  |  |  |
|  | CAST16 (R) | .30 | < .001 |  |  |  |
|  | CAST17 | .63 | < .001 |  |  |  |
|  | CAST18 | .43 | < .001 |  |  |  |
| Self-enhancing humor | SEH1 | .65 |  | .90 | .12 | .06 |
|  | SEH2 | .45 | < .001 |  |  |  |
|  | SEH3 | .88 | < .001 |  |  |  |
|  | SEH4 | .67 | < .001 |  |  |  |
|  | SEH5 | .86 | < .001 |  |  |  |
|  | SEH6 (R) | .49 | < .001 |  |  |  |
|  | SEH7 | .57 | < .001 |  |  |  |
|  | SEH8 | .34 | < .001 |  |  |  |
| Affiliative humor | AffH1 (R) | .58 |  | .92 | .10 | .05 |
|  | AffH2 | .62 | < .001 |  |  |  |
|  | AffH3 (R) | .47 | < .001 |  |  |  |
|  | AffH4 | .64 | < .001 |  |  |  |
|  | AffH5 (R) | .69 | < .001 |  |  |  |
|  | AffH6 | .66 | < .001 |  |  |  |
|  | AffH7 (R) | .54 | < .001 |  |  |  |
|  | AffH8 (R) | .63 | < .001 |  |  |  |
| Aggressive humor | AggH1 | .48 |  | .86 | .08 | .06 |
|  | AggH2 (R) | .36 | < .001 |  |  |  |
|  | AggH3 | .38 | < .001 |  |  |  |
|  | AggH4 (R) | .60 | < .001 |  |  |  |
|  | AggH5 | .39 | < .001 |  |  |  |
|  | AggH6 (R) | .57 | < .001 |  |  |  |
|  | AggH7 | .45 | < .001 |  |  |  |
|  | AggH8 (R) | .58 | < .001 |  |  |  |
| Self-defeating humor | SDH1 | .65 |  | .95 | .08 | .04 |
|  | SDH2 | .81 | < .001 |  |  |  |
|  | SDH3 | .69 | < .001 |  |  |  |
|  | SDH4 (R) | .36 | < .001 |  |  |  |
|  | SDH5 | .68 | < .001 |  |  |  |
|  | SDH6 | .57 | < .001 |  |  |  |
|  | SDH7 | .35 | < .001 |  |  |  |
|  | SDH8 | .72 | < .001 |  |  |  |
|  |  |  |  |  |  |  |

Descriptive Statistics for Means Without Low Loading Items

| **Table 2**  *Sample Descriptives of Means with low Loading Items Excluded.* | | | | | |
| --- | --- | --- | --- | --- | --- |
|  | *M* | *SD* | Min | Max | Potential range |
| Age | 26.46 | 5.88 | 18 | 77 | 18 – 99 |
| Global trolling | 1.36 | 0.50 | 1 | 4.40 | 1 – 5 |
| Immediate trolling motivation | 1.08 | 0.29 | 1 | 3.80 | 1 – 5 |
| Machiavellianism | 2.56 | 0.75 | 1 | 4.83 | 1 – 5 |
| Narcissism | 2.67 | 0.70 | 1 | 5.00 | 1 – 5 |
| Psychopathy | 1.85 | 0.62 | 1 | 4.14 | 1 – 5 |
| Sadism | 1.46 | 0.47 | 1 | 4.06 | 1 – 5 |
| Aggressive humor | 2.92 | 1.08 | 1 | 6.20 | 1 – 7 |
| Affiliative humor | 5.81 | 0.83 | 1.88 | 7.00 | 1 – 7 |
| Self-enhancing humor | 4.62 | 1.11 | 1 | 7.00 | 1 – 7 |
| Self-defeating humor | 3.11 | 1.24 | 1 | 7.00 | 1 – 7 |
| *Note*. *n* = 1,026 | | | | | |

Correlation Matrix for Means Without Low Loading Items

| **Table 3**  *Correlation Matrix for Trolling, the Dark Tetrad of Personality, and Humor Styles Using Means with low Loading Items Excluded.* | | | | | | | | | |
| --- | --- | --- | --- | --- | --- | --- | --- | --- | --- |
|  | 2. | 3. | 4. | 5. | 6. | 7. | 8. | 9. | 10. |
| 1. Global trolling | .46** | .37** | .31** | .46** | .49** | .33** | -.01 | .05 | .14** |
| 2. Immediate trolling motivation | 1 | .20** | .19** | .33** | .40** | .27** | .001 | -.05 | .14** |
| 3. Machiavellianism |  | 1 | .36** | .66** | .49** | .36** | -.07* | .02 | .17** |
| 4. Narcissism |  |  | 1 | .40** | .38** | .27** | .17** | .28** | .08* |
| 5. Psychopathy |  |  |  | 1 | .62** | .46** | -.08** | .004 | .22** |
| 6. Sadism |  |  |  |  | 1 | .53** | -.03 | .05 | .17** |
| 7. Aggressive humor |  |  |  |  |  | 1 | -.03 | .09** | .21** |
| 8. Self-enhancing humor |  |  |  |  |  |  | 1 | .37** | .05 |
| 9. Affiliative humor |  |  |  |  |  |  |  | 1 | .08** |
| 10. Self-defeating humor |  |  |  |  |  |  |  |  | 1 |
| *Note*. *n* = 1.026. ** *p* < .01 (two-tailed) | | | | | | |  |  |  |

*t*-Test for Trolling Motivation Without Low Loading Items

To test our third hypothesis (H3: Participants who are socially excluded show increased immediate trolling motivation compared to people who are socially included), we conducted a *t*-test with exclusion (yes/no) as the independent and immediate trolling motivation as the dependent variable. Our result suggests that the experience of exclusion did not significantly impact participants’ immediate trolling motivation, *t*(1024) = 0.36, *p* = .72, CI = [-.03; .04]. Thus, findings from this analysis did not support H3.

Quantile Regression for Means Without Low Loading Items

| **Figure 1**  *Graphs of the Quantile Regression Coefficients for all Independent Variables of Means with Low-Loading Items Excluded.* |
| --- |
|  |
| 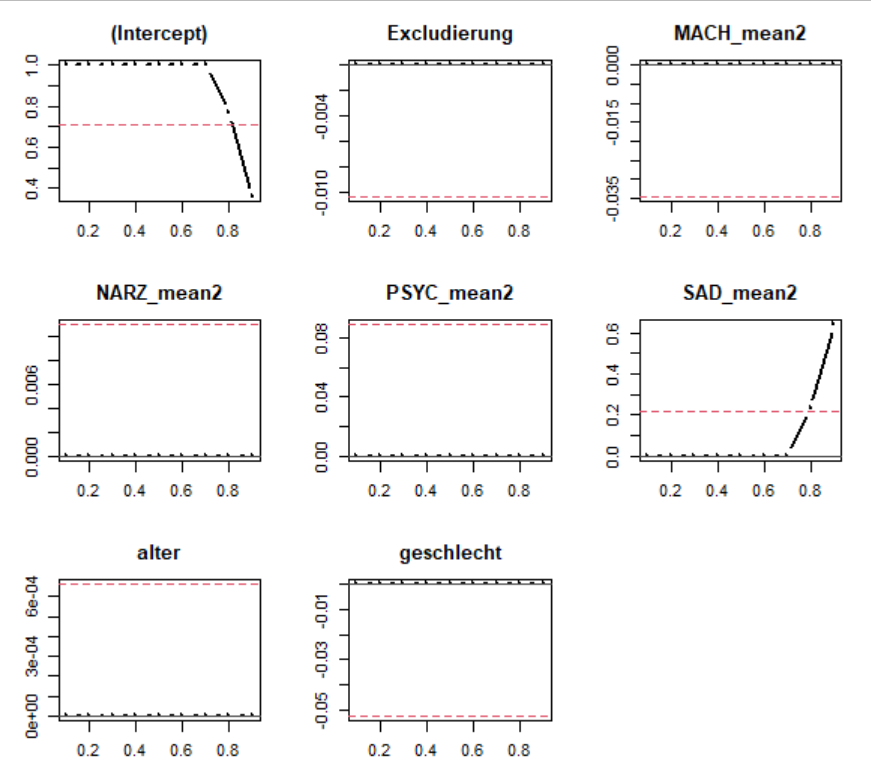 |
| *Note*. Simple linear regression coefficients (red line) and quantile regressions for exclusion, Machiavellianism, narcissism, psychopathy, sadism, age, and gender (male with female as the comparison group) for the dependent variable immediate trolling motivation. The x-axis represents the quantiles for immediate trolling motivation while the y-axis represents the unstandardized coefficients of the respective independent variable. |

| **Table 4**  *Quantile Regression Coefficients of Exclusion, Machiavellianism, Narcissism, Psychopathy, Sadism, Age, and Gender for the Dependent Variable “Immediate Trolling Motivation” with Low Loading Items Excluded from Means.* | | | | | | | | |
| --- | --- | --- | --- | --- | --- | --- | --- | --- |
|  | Quantile regression coefficients | | | | | | | |
| Quantiles | Intercept | Exclusion | Machiavellianism | Narcissism | Psychopathy | Sadism | Age | Male |
| 0.10 | 1*** | 0 | 0 | 0 | 0 | 0 | 0 | 0 |
| 0.20 | 1*** | 0 | 0 | 0 | 0 | 0 | 0 | 0 |
| 0.30 | 1*** | 0 | 0 | 0 | 0 | 0 | 0 | 0 |
| 0.40 | 1*** | 0 | 0 | 0 | 0 | 0 | 0 | 0 |
| 0.50 | 1*** | 0 | 0 | 0 | 0 | 0 | 0 | 0 |
| 0.60 | 1*** | 0 | 0 | 0 | 0 | 0 | 0 | 0 |
| 0.70 | 1*** | 0 | 0 | 0 | 0 | 0 | 0 | 0 |
| 0.80 | 0.77^***^ | 0 | 0 | 0 | 0 | 0.23* | 0 | 0 |
| 0.90 | 0.36** | 0 | 0 | 0 | 0 | 0.64*** | 0 | 0 |
|  | OLS regression coefficients (SE) [BCa-95% CI] | | | | | | | |
|  | Intercept | Exclusion | Machiavellianism | Narcissism | Psychopathy | Sadism | Age | Male |
|  | .71 (.06) [.55; .86] | -.01 (.02) [-.05; .02] | -.03 (.02) [-.08; .001 | .01 (.01) [-.01; .04] | .09 (.02) [.04; .14] | .22 (.03) [.11; .31] | .001 (.001) [-.002; .004] | -.05 (.02) [-.14; .04] |
| *Note*. ^t^ *p* < .10. * *p* < .05. ** *p* < .01. *** *p* < .001. OLS = ordinary least square. | | | | | | | | |

Dominance analysis

Analysis using Traditional Means

Overall, the results suggest that sadism and psychopathy contribute most to explaining immediate trolling motivation while age, gender, and exclusion experience contribute the least. Our dominance analysis indicates a general dominance of sadism (0.09) and psychopathy (0.09) over the remaining variables (< 0.02). Both variables completely dominate over social exclusion experience, Machiavellianism, narcissism, age, and gender.

Analysis using Means Without Low-Loading Items

When conducting the dominance analysis using the means without low-loading items, the results slightly shift: Now, sadism dominates all other predictor variables, including psychopathy. The general dominance of sadism increases to 0.10 while the dominance of psychopathy reduces to 0.05. The remaining variables have average contributions of less than 0.01. Sadism completely dominates over psychopathy. This, like our quantile regression, highlights the importance of sadism in predicting trolling behavior.
