# Supplementary material for: Troll story: The dark tetrad and online trolling revisited with a glance at humor
Source: PLoS One. 2023 Mar 10;18(3):e0280271. doi: 10.1371/journal.pone.0280271 (PMC10004561; doi:10.1371/journal.pone.0280271)
Supplement: Attachment — Submitted filename: PlosONE review 1.docx [file pone.0280271.s003.docx]

**PlosONE review**

Manuscript: PONE-D-22-17599; Troll Story: The Dark Tetrad and Online Trolling Revisited with a Glance at Humor

I would like to congratulate the authors on a splendid reworking of their article! All my comments (and more!) have been satisfactorily addressed and I am pleased to recommend this paper for publishing.

The section on katagelasticism was particularly enjoyable. The intention behind studying the Dark Tetrad traits, trolling, and humour styles together is a lot clearer. Thank you for addressing concerns about the measures that were used.

The only minor error I picked up on was under Section 3.4 “the first graph in Figure 1 shows how Machiavellianism…”. It looks like it would be the either the first row or third graph depicting Machiavellianism. The same section (3.4) has its title in bold, which should be reformatted to normal.

Once again, thank you for taking the time to comb through the manuscript, making all the necessary changes, and adding in more recent citations.
