# Supplementary material for: Troll story: The dark tetrad and online trolling revisited with a glance at humor
Source: PLoS One. 2023 Mar 10;18(3):e0280271. doi: 10.1371/journal.pone.0280271 (PMC10004561; doi:10.1371/journal.pone.0280271)
Supplement: Attachment — Submitted filename: Response to Reviewers.docx [file pone.0280271.s004.docx]

**PlosONE review**

Manuscript: PONE-D-22-17599; Troll Story: The Dark Tetrad and Online Trolling Revisited with a Glance at Humor

**Reviewer #3:** The only minor error I picked up on was under Section 3.4 “the first graph in Figure 1 shows how Machiavellianism…”. It looks like it would be the either the first row or third graph depicting Machiavellianism. The same section (3.4) has its title in bold, which should be reformatted to normal.

**Response by authors:** We would like to thank Reviewer #3 for their kind feedback and keen eye. The section heading of 3.4. has been reformatted to normal. We have also fixed the error of referring to the Machiavellianism graph: “the third graph in the first row of Figure 1 shows how Machiavellianism …”
